# The internal realities of individuals with type 2 diabetes  – a functional framework of self-management practices via Grounded Theory approach

**DOI:** 10.1371/journal.pone.0225534

**Published:** 2019-11-26

**Authors:** Yogarabindranath Swarna Nantha, Shamsul Haque, Azriel Abisheg Paul Chelliah

**Affiliations:** 1 Primary Care Research Initiative And Methods Education Department (PRIMED), Seremban Primary Health Care Clinic, Seremban, Malaysia; 2 Non-Communicable Disease Department, Seremban Primary Health Care Clinic, Seremban, Malaysia; 3 Department of Psychology, Monash University Malaysia, Subang Jaya, Malaysia; Chinese Academy of Medical Sciences and Peking Union Medical College, CHINA

## Abstract

**Background:**

An upward trend is observed in the prevalence of Type 2 diabetes (T2D) in South-East Asian and Western Pacific regions. These patterns incur a costly health burden to developing nations around the world. A clear understanding of the mechanics behind self-management practices of T2D patients might help overcome this impasse. This information can help unlock specific problem areas that warrant specific intervention. We aim to uncover prevailing T2D self-management habits and its deviations from optimal behaviour.

**Methods:**

We adopted a Grounded Theory approach to guide in-depth interviews (IDI) with T2D patients and healthcare providers (HCP) at a regional primary care clinic in Malaysia. Twenty-four T2D patients and 10 HCPs were recruited through purposive sampling to examine their inner psychological narratives related to self-management practices. 2 focus group discussions (FGD) were conducted as a part of the data triangulation process.

**Results:**

A functional framework for self-management practices in T2D patients was developed. Self-management behavior was characterized by 2 major processes– 1) helpful and, 2) unhelpful practices. Self-efficacy, taking responsibility and being rational define helpful behaviour in these patients. On the other hand, unhelpful traits (neglect, poor restraint, and experimentation) often trigger violations with regards to medication compliance and therapeutic lifestyle changes.

**Conclusions:**

We outlined a roadmap that navigates through the positive and negative mindset in relation to self-management practices of T2D patients. These results highlight the importance of devising individualized strategies by taking into account the personal challenges, emotions, and motivations that define the inner self of the patient.

## Introduction

In 2017, approximately 451 million individuals were estimated to be living with diabetes worldwide[1]. This figure is projected to increase up to 693 million cases in the year 2045[1]. These numbers do not take into account a large reservoir of undiagnosed cases that fall within the South-East Asian and Western Pacific regions[1]. Alarmingly, 5 million deaths across the globe are attributed to the complications of diabetes[1]. The combination of these scenarios imposes a huge burden on the world’s total health expenditure, which is also expected to increase to a hefty USD 958 billion by the year 2045[1].

Diabetes is largely preventable[2]. Epidemiological studies demonstrate that primary prevention initiatives could potentially reduce the risk of all-cause and cardiovascular risk up to 9.0%[3]. Therefore, experts believe that a purely “glucocentric” approach to managing persons with T2D is no longer an effective technique to change the tide against the rising trends of diabetes[4–5]. In accordance with the principles of preventative management, a holistic therapeutic approach is much needed in people with well-established T2D[2]. This strategy necessitates comprehensive changes to be made to lifestyle habits, exercise patterns, dietary measures, psychological wellbeing, compliance to medications, self-monitoring, problem-solving skills, and risk reduction behaviours[2–7].

Behavioural change and disease self-management are the cornerstones of optimal glycaemic control in people with T2D[8].To date, this remains the Gordian knot that needs to be untied to usher in a paradigm shift to the way we understand T2D management[9].The solution to this conundrum is defined by its complexity–T2D management involves the unraveling of the qualities of self, personal identity and the living experiences of people with T2D[9]. Hence, it is imperative to understand innate self-management practices by first identifying the salient determinants of this behaviour “within” T2D patients[9]. To that end, the overarching connections between these qualities need to be clearly delineated through theory-driven research endeavours[10].

This article expounds the emergent themes (S1 Table and S2 Table) related to self-management practices that were obtained from a more comprehensive study we conducted using a Grounded theory approach[11]. We aim to 1) investigate pre-existing self-management practices in T2D patients, 2) identify deviations from expected optimal behaviour,3) classify the intricacies of their behaviour into actionable categories and 4) present an operational self-management model. We also seek to verify the results of this study through the rigorous process of triangulation. This step involves data collection from multiple sources, persistent observation, negative case analysis, and consensus decision-making.

## Material and methods

### Design

A qualitative approach was adopted to elucidate detailed behavioural patterns pertinent to the objectives of this study. An explorative inquiry with an inductive approach (S2 Table) was employed to 1) discover the inner psychological disposition of individuals with T2D, 2) generate inferences based on emergent data, and 3) use theory-building techniques as the foundation for newer insights other than those documented in literature[12].

### Participants and setting

Selected T2D people were recruited using a purposive sampling method from the non-communicable disease department at the Seremban Primary Care Center, a regional multidisciplinary general practice clinic within the state of Negeri Sembilan, Malaysia. A heterogeneous sample was acquired through a maximum sampling strategy. This step allowed the enrolment of participants with varying levels of socioeconomic and disease control indicators in order to improve the generalizability of our study (Table 1 and Table 2). A previously published paper provides detailed information about the research methods employed in this study while demographic data of the participants are depicted in Tables 1 and 2[11].

**Table 1:** Demographic details of T2D participants of the study.

Subject Characteristics	*N*
	
**Gender**	
Male	15
Female	9
**Age In Years**	
< 30	1
31–50	6
51–60	7
61–70	7
> 70	3
**Ethnicity**	
Malay	12
Chinese	1
Indian	10
Others	1
**Marital Status**	
Unmarried	3
Married	19
Divorced	1
Widow/Widower	1
**Work Status**	
Not working	12
Working Full-time	8
Working Part-time	1
Self-employed	3
**Level Of Education**	
Certificate level	19
Diploma	3
Bachelor’s degree	2
**Diabetes duration (years)**	
< 5	5
6–10	3
11–15	4
16–20	2
> 21	3
**Number of medications**	
3–5	12
> 6	12
**Type of medication**	
Oral only	5
Insulin only	4
Combination	15
**Glycaemic status**	
6.5–8.0%	3
8.1–9.0%	9
9.1–10%	4
> 10%	8
**Complications**	
No	17
Yes	7
**Number of comorbidities**	
1	13
2	4
> 3	8

**Table 2:** Demographic details of health care workers of the study.

Subject Characteristics	*N*
**Profession**	
Diabetic educator (nurse)	5
GPs	3
Pharmacists	2
**Mean Age In Years**	34
**Mean Years In Service**	10
**Ethnicity**	
Malay	7
Chinese	1
Indian	2
**Marital Status**	
Unmarried	2
Married	7
Divorced	1
**Level Of Education**	
Diploma	3
Bachelor’s degree	5
Master’s degree	2

### Data collection

Thirty-four people with T2D and 14 healthcare providers (HCP) were invited to participate in the study. Four HCP declined to participate and 10 persons with T2D did not respond to our invitation. Therefore at the point of theoretical saturation, 24 people with diabetes and 10 HCP eventually took part in in-depth interviews conducted between May 2018 and February 2019. Each interview lasted between 75 to 90 minutes. Subsequently, 2 FGDs (8 T2D participants in each session, each session lasting approximately 90 minutes) were conducted as part of the data triangulation process.

Participants were interviewed in a designated research room within the premises of the clinic. A previously published study protocol explains details related to the interviewing technique and the topic guide employed in this study[11]. Attempts were made to minimize the framing of questions that were influenced by the interviewer’s personal beliefs by permitting participants to express issues perceived highly salient to them. No additional new themes or categories were obtained after the 10^th^round of interviews. Thus, theoretical saturation was considered to have been achieved at this point. However, the establishment of the validity of these categories required additional interviews with 14 persons with T2D where these narratives were further explored, enriched, and consolidated. The interviews were audiotaped and transcribed verbatim. One researcher (YSN) conducted all interviews.

### Data management and analysis

All analyses were conducted in both Malay and English language in order to preserve contextual validity. Interviews conducted in the Malay language were transcribed without any translation. Quotations were read in its original language and coded in English. Quotations in the Malay language were translated into English for publication purposes.

All transcripts were proofread by the third author and was re-examined by the first author for completeness. Subsequently, the proofread transcripts were entered into a qualitative software (Atlas.TI qualitative analysis program, Version 7, Cincorn Systems Inc, 2008) and coded manually by the first author to identify specific themes. The data was coded in accordance with the classical and constructivist Grounded Theory methods[12,13].Frequent discussions were held between the second and third author to refine the emerging themes throughout the process of data analysis.

Transcripts were coded independently and a list of emerging themes and categories were generated. Concurrently, two independent coders (AAPC and GKY) examined and coded the transcripts for categories, themes and sub-themes. Disagreements were resolved in discussion comprising authors and independent advisors (CMHC, HK, LXY, LWY, GA). As a result, excellent inter-coder consistency (Cohen’s κ = 0.84) and expert panel consensus were found (Cohen’s κ = 0.88).

### Ethical consideration

This study received approval from the Medical Research and Ethics Committee, Ministry of Health Malaysia (NMRR-18-151-39886) on the 30th of May 2018 and also Monash University Human Research Ethics Committee on 10^th^ of October 2018 (Project ID: 17062).The details of the study were conveyed to the participants personally by the researcher prior to IDIs and FGDs. Additionally, participants were also provided with an information sheet containing the procedures that will be undertaken in the study. Subsequently, we obtained written consent and permission from all participants to audio record the interview sessions.

## Results

**Fig 1:**
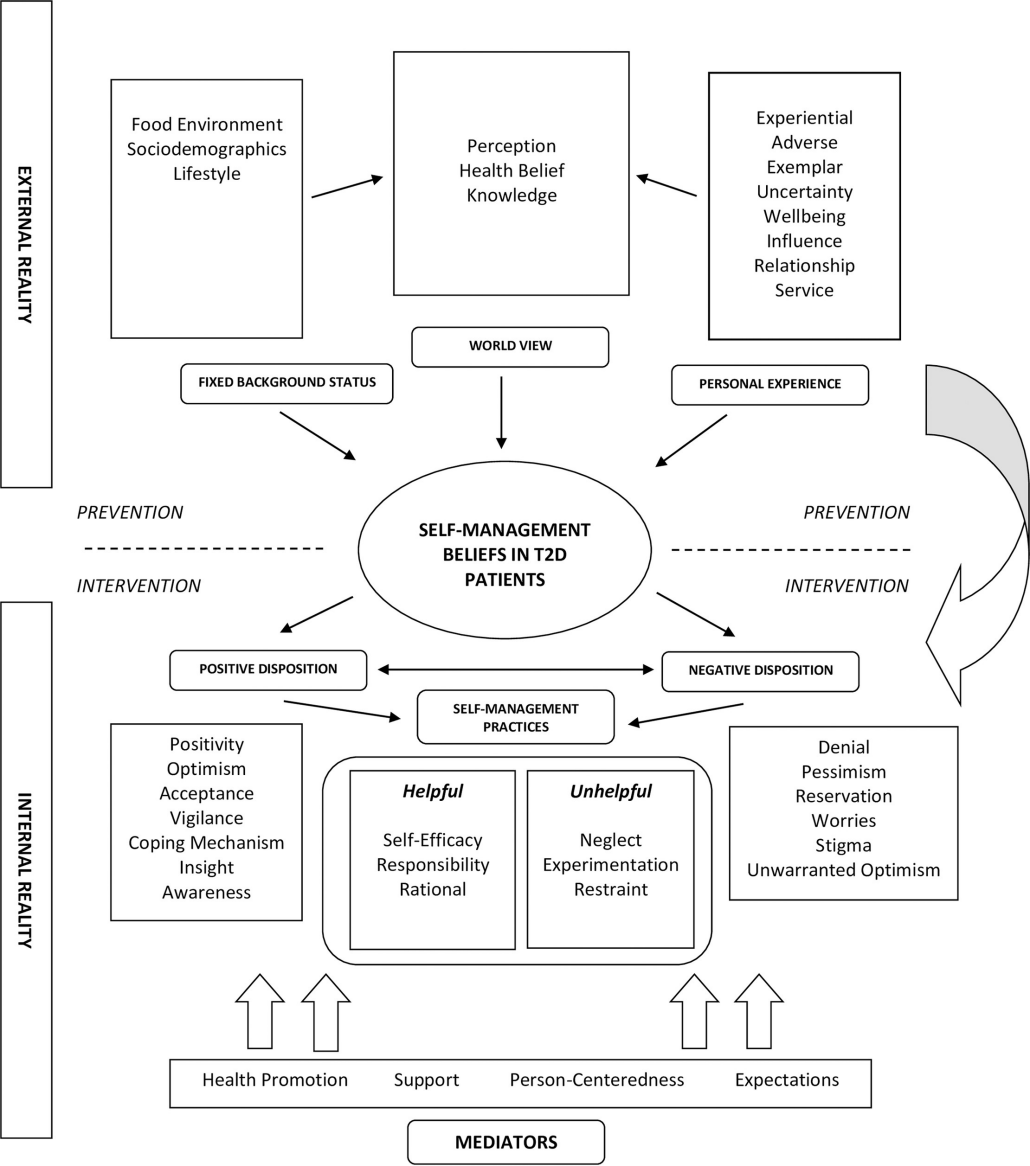
Conceptual framework describing the external reality, internal reality and mediators related to self-management of T2D.

The dynamic interaction between each dimension that define the experience of living with T2D is exemplified by the conceptual framework depicted in Fig 1. In this manuscript, we will limit our description to 2 major themes that determine the active management of the disease and the sustenance of these actions (Table 3).These themes depict self-care behaviour in T2D people and can be classified into 2 broad categories–helpful (self-efficacy, rationality, responsibility) and unhelpful (neglect, experimentation, poor restraint) self-management practices.

**Table 3:** Categories, definitions, frequency, and codes describing self-management practices of T2D patients.

Category	Sub-Category	Frequency	Codes
**Helpful Self-Management Practices**	Self-Efficacy	386	Compliance with medications
			Positive dietary measures
Positive actions taken to manage symptoms, treatment and lifestyle changes			Personal goals
	Responsibility	253	Being proactive about disease
			Having self-discipline
			Need to support family
			Compliance with medications
	Rational	223	Thinking and processing information related to treatment
			Role of complementary alternative medications
			Trusting opinion of others
**Unhelpful Self-Management Practices**	Neglect	287	Factors related to non-compliance of medications
			Poor dietary and exercise compliance
	Poor restraint	152	Craving
			Dietary violations despite awareness
	Experimentation	108	Experimenting with medication regime
			Relying on complementary alternative medications

### Helpful self-management practices

#### Self-efficacy

A large majority of T2D people confess complying with the recommended medication regime. To a certain extent, they adhere to instructions inscribed on their prescription slip especially specific information related to medication intake timings. Interestingly, T2D people also invoke convenient mental reminders in the form of subtle cues by constantly “telling one’s self that we need to take medications”. Many others adopt key lifestyle changes that eventually translate into actual physical action. One powerful example of this behaviour is the creation of a regular ritual that integrates the act of medication intake as “a part of their daily routine”. GPs, nurses, and pharmacist agree that T2D patients actively take steps to mitigate the occurrence of a missed dose by making several modifications to their pre-existing lifestyle habits which include 1) setting reminders on their handphones, 2) storing medications in containers, 3) bring along medications when out of home and, 4) keeping some amount of medications at their workplace.

The prime motivation to continue taking their medications is driven by the desire to prevent complications that occur as a result of uncontrolled T2D. To this end, T2D people regularly attend scheduled consultations and develop the need to consult the GPs whenever any doubt arises. They also practice proper foot care and recall the accurate technique of insulin utilization (e.g. insulin administration and storage). There is a greater tendency to use a glucometer device for self-monitoring purposes in both insulin users and T2D patients who solely use oral medications. Readings from glucometer devices provide real-time feedback of their glycaemic status. This step leads to improvements in self-care, especially in terms of medication compliance and dietary adherence.

“I frequently check my blood glucose levels [using a glucometer]. If the reading exceeds 9 or 10, I’ll start asking myself what I ate on that particular day.” (IDM 011)

“If we comply with medications as recommended, I am certain of its efficacy. I have some of those glucometer strips with me, so I can test it [blood glucose levels] whenever I want to. So, when I don’t consume my medications the readings go up as high as 18 or 20. But when I take them regularly, it remains at 6 or 7.” (IDM 023)

A large proportion of individuals with T2D claim they follow dietary measures advocated by HCPs after an initial period of adaptation. They choose to consume more vegetables and often reduce the portion of daily rice intake. They also practice restraint when consuming their daily diet (i.e. restricting sugar intake). Persons with T2D acknowledge being able to still enjoy food despite reducing the amount previously consumed. With the passage of time, they develop an innate mindset that reinforces the need to control their diet.

“To what degree do you want to eat something delicious all the time? Why do you need to indulge in that kind of behaviour? You know, I have reached that realization where I believe there’s no end to satisfying your taste buds. We are all human after all. So, I keep telling myself that it’s completely up to me to change myself.” (IDM 021)

More weathered T2D patients begin to develop personal goals revolving around the wish of bringing down their blood glucose readings to more acceptable levels. To achieve this, they engage in constructive lifestyle changes through exercise and dietary measures. They show an open willingness to maintain healthy “eating patterns” in a bid to achieve better T2D control. Similarly, regular exercise is viewed as a compulsory adjunct to medications that has the potential to stabilize overall glycemic status.

“I try to ‘reduce’ this sickness by doing frequent exercises. I don’t think it will completely cure my diabetes but I am sure there will be some improvements. In my mind, I believe maybe by exercising, more sugar will be depleted from my body” (IDM 003)

#### Rationality

People with T2D frequently scour the internet in search of information related to disease management. A great deal of thinking goes into making a conscious decision of choosing what information is useful to them. To a certain degree, they trust the information obtained through the internet, especially if it has some scientific or evidence-based merit to it. Only a minority of persons with T2D remain cautious and skeptical about information on the internet, stating that certain websites spread misinformation about disease management.

“If you go on the internet and look at all those websites, they tell you a lot of stories about how diabetes can be reversed, that you don’t need to take any medication. So, people tend to believe it and they do all kinds of things thinking it’s a curable disease.” (IDM 010).

Although they receive constant feedback from society about how to manage their disease, people with T2D know that it is ultimately “up to them” to accept the opinion of others. They place greater trust in the advice dispensed by change agents such as doctors. Nevertheless, they feel that the onus is on the individuals with T2D to make that judgement call and to follow through on the instructions given by GPs.

“It’s their [patient] body and their choice [to follow advice]. But it is your [GPs] duty to give them [T2D patients] advice. It’s up to them if they want to take up on the advice or not.” (IDM 011)

A large proportion of participants remain skeptical about the role of complementary alternative medication (CAM) in the treatment of T2D. They dismiss the need for CAM and place more faith in conventional medications which have been proven to be far more effective. A small number of T2D people doubt the efficacy of CAM after personally having used it in the past. GPs, pharmacist, nurses, and T2D patients fear CAM intake could lead to unforeseen adverse effects. Hence, there is a general consensus that CAM should only be trusted if it is supported by scientific evidence.

#### Responsibility

People with T2D agree that “disciplining one’s self” is the cornerstone of optimal T2D management. To them, self-discipline is an important personal quality that influences their determination to adhere to medication intake, dietary advice or exercise activities. T2D people feel they “have only themselves to blame” for not being responsible enough, especially with regards to medication compliance.

Taking ownership of one’s disease appears to be an important belief shared by many HCPs and individuals with T2D during interviews. This belief is characterized by the need to adopt a more proactive stance towards their disease. For instance, persons with T2D often inquire GPs about the prognosis of their disease and do their own research to discover information about various T2D treatment modalities.

Many T2D people manage their disease independently and do not rely on family members for help. They realize that “it is their responsibility” to “look after their own health” if they wish to live longer and have a better quality of life. They want to be healthy so that they can continue to support the welfare of their family. More importantly, they want to avoid being a burden to family members in the event they become an invalid as a result of their disease. Therefore, the act of taking medications is central to their health maintenance in the long run.

“I can’t emphasize enough the need to take control of our disease. You see, the doctor is not going to check your feet or your eyes every day. You see them only after 4 months or so. We have to look after ourselves. We need to rely on ourselves.” (IDM 018)

“Taking medications is one way of keeping ourselves healthy” (IDM 004)

### Unhelpful self-management practices

#### Neglect

GPs, pharmacist, nurses and T2D people alike concur that the major cause for poor disease control is the lack of self-discipline to follow through on T2D management. In the same way, some T2D people adopt a lackadaisical attitude towards disease management which is often characterized by a pervasive “taking it easy” mentality. Therefore, it is not uncommon to observe 1) deliberate non-compliance to medications, 2) violations in the proper timing for medications intake, and 3) violations in proper insulin techniques.

“There are many reasons why diabetes is a norm. For example, people often crave for food and adopt the usual ‘I don’t care’ sort of attitude.” (IDM 011)

“I get my prescription from the doctor and collect the medication as always, but when I get home, I go slide back into my old ways of going about my disease.” (IDM 015)

“You can control your disease by taking medication, dieting and exercising regularly. However, I don’t do all those things.” (IDM 009)

In terms of therapeutic lifestyle changes, they avoid commitments that require them to adhere to dietary restrictions and confess not getting enough exercise. During consultations, some people with T2D fail to disclose vital clinical information about themselves to the GP (e.g. detecting predominantly high blood glucose readings at home) which would have facilitated a more appropriate management of their disease. Ironically, despite all their conscious acts of underperformance, T2D patients remain acutely aware that they are to be held personally accountable for these shortfalls in self-care practices.

“Sometimes, it’s my mistake. I don’t follow the doctor’s advice. Especially with regards to food intake.” (IDM 002)

“I feel that, if I ever suffer any complications [from T2D], for example getting my foot amputated, it would be entirely my fault. I know because I didn’t do enough to control my disease effectively.” (IDM 023)

Individuals with T2D cite “unfamiliarity” with T2D as a reason for their apparently detached attitude towards the seriousness of their disease. Prior to the diagnosis of T2D, they felt “perfectly normal” to the extent that they were completely unaware that they actually have T2D. To them, the chances having of T2D often remains a remote possibility and only extreme physical symptoms (e.g. dizziness or physical pain) serve as a potent reminder to the immediacy of their disease. Upon receiving the diagnosis of their disease, they believe medications are not meant to be taken for the rest of their lives, with a chance of weaning them off later in the future. Without any apparent physical symptoms to alert them about their disease, they develop ambivalence to complying with their medications. Even after living with the disease for many years, some individuals with T2D still remain ignorant about the consequences of uncontrolled T2D. They appear to only comply with proper T2D management once complications have set in.

T2D people condone missing their medications “just a little”, declaring that such episodes are just “acceptable mishaps”. They cite “forgetfulness” as a common reason for missing their medications—they consider this behaviour as a normal part of the aging process. This fact was further corroborated by interviews with both pharmacists and nurses. Many T2D people attempt to circumvent this problem by setting reminders on their handphones but to no avail. In the course of the day, persons with T2D often procrastinate consuming their medications. This behaviour causes them to eventually miss their medications entirely when they become either too engrossed in their work or as a result of a hectic daily routine. A more worrisome phenomenon is the intentional act of skipping medications. One individual with T2D skipped his medication deliberately, saying “it takes far too long for these medications to work”. The urgency of taking their medications only occurs to them when the date of their appointment with the GP looms closer.

“Whenever you see the GP, you’ll be motivated to take your medications for a month or two. When it gets closer to the visit to the GP, you get apprehensive. You say to yourself ‘oh boy, I’ve got an appointment and I got to follow my doctor’s advice’.” (IDM 012)

#### Poor restraint

T2D people attribute the development of T2D to uncontrolled food consumption. Prior to their disease, they lacked awareness about the dangers of a sugar-laden diet. In more experienced T2D people, recidivism associated with dietary non-compliance is primarily linked to a combination of poor restraint and food cravings. IDIs demonstrate their frustrations of adhering to dietary restrictions which is evidenced by the difficulty they face in controlling their diet. They are also easily susceptible to dietary violations when under social pressure.

“Poor dietary control is the main source of all my problems. When I am with friends at the restaurant, I see them ordering sweetened tea and fried noodles. I can’t accept that reality doctor–if they can eat all that, so can I, I have money too. And then off I go, ordering and consuming the same food they eat.” (IDM 015)

They exhibit craving-like tendencies by consuming food in excess and often consume food that are clearly forbidden despite knowing better. They condone these “temptations” by stating that it is just an infrequent deviation from their normal dietary habits.

“Maybe once in a while doctor, especially when we go out, there’s always that temptation [violating dietary restrictions]” (IDM 017)

#### Experimentation

Experimentation (e.g. skipping or altering medication dose) in the way T2D patients consume their medications is a relatively common finding. Individuals with T2D persist taking a dosage that is different from what has been suggested by their GPs. Thus, they invariably witness a surge in their blood glucose levels due to the consumption of a sub-theraupeutic dose of their medications.

A vast majority of T2D people disclose that they tend to use CAM especially if it originates from plant-based sources such as herbs, spices or vegetables. They do not substitute conventional medications (although very few individuals with T2D do) with CAM but use it temporarily alongside CAM as a supplement to “bring down” an unexpected upsurge in blood glucose levels. Fearing unforeseen effects of the concurrent usage of CAM and prescribed drugs, T2D people establish a time interval to “buffer the metabolism” between these medications.

“We can’t take both together [CAM and prescribed medications]. We must allow a delay of at least half an hour. We must maintain that time interval so that medications will work [effectively] in our body.” (IDM 009)

## Discussion

### Summary of main findings

Our study depicts the naturalistic manner in which most T2D people operate in relation to their self-management behaviour. In principle, the conceptual framework proposed in Fig 1 suggests that there is an unyielding ideological boundary between the helpful and unhelpful volitional traits seen in these individuals with T2D. However, the evidence from our interviews indicate that this boundary is essentially arbitrary. Therefore, the inherent nature of self-management practices lies between a vibrant continuum where both positive and negative actions can coexist albeit in different intensities.

The naivety about their disease under the guise of being ignorant about the consequences of their disease appears to dominate the psyche of a large number of people with T2D. To them, this evident lack of intrinsic motivation to look after themselves is “permissible” and is partly reinforced by their “relaxed” attitude towards the disease. On the other hand, more “responsible” T2D people employ metacognition as a way to regulate self-care practices[14]. These individuals view the competence of disease management as being proportional to the conscious act of reminding one’s self to look after their health. Consequently, this behaviour attenuates neglect and nurtures more positive behaviour such as self-efficacy or responsibility.

### Strengths and limitations

The major strength of this study lies in the utility of the naturalistic self-management model described in Fig 1. Primarily, this model provides a functional framework from which one could test certain hypotheses related to the internal realities and the external environment that govern disease self-management practices in T2D patients (Fig 1, S1 Table & S2 Table). Secondly, the framework delineates several clinically applicable domains that might help in the development of psychological inventories within the field of self-management research. Thirdly, the model identifies factors pertinent to the implementation of a person-centered intervention in disease self-management that is extremely vital to any routine medical consultation. The integration of these components reflect a holistic conceptualization of self-management practices within the context of the living environment of a T2D person.

We narrowed our research focus to only investigating the mindset of individuals with T2D from a primary care setting. Although it is equally important to assess the input from participants from other clinical settings, these findings are highly relevant to GPs, whom, customarily devote a considerable amount of time addressing psychosocial issues highlighted in this study. Nevertheless, we strove to enhance generalisability of our findings by obtaining participants 1) from a community-based setting and 2) with a mixed sociodemographic background (Tables 1 and 2).

We recruited T2D patients from a diverse cultural, economic, social and clinical background. This strategy could have had an impact on the representativeness of the psychological narrative derived from our study. However, we found that the views held by different subgroups of T2D patients (based on sociodemographic or clinical characteristics) remained congruent and did not diverge much from the thoughts shared by other participants. Moreover, the evidence from our interviews (especially during FGDs) imply that most T2D patients often embody a consistent set of beliefs in relation to their self-management practices. For example, the utilization of insulin or inherent complications only influences the polarity of their belief system that is within the scope of the behavioural dimensions described in our model (Fig 1).

### Comparison with literature

This study depicts the summation of the various behavioural traits of the archetypal T2D people into one single operational model. Contrary to previous fragmentary descriptions of self-management practices in the form of systematic reviews or qualitative metanalysis[15–17], our study emphasizes on the connections and interactions that exist between these behavioural attributes of a typical T2D person found at a conventional clinical setting. While many of the themes presented in this study have been described elsewhere in literature[15–17], our study collates these findings into a functional model that define the attributes of the individual T2D person with regards to self-management practices (Fig 1).

We found adherence to medication regimen is the most important self-management activity undertaken by individuals with T2D. This finding is consistent with the data collated from various studies investigating self-care activities in diabetes care[18]. Similarly, many T2D people in our study recommend creating a routine to medication administration times, declaring that this a vital step in the maintenance of proper medication compliance[19]. More importantly, we noticed that people with T2D who exhibit medication avoidance also demonstrate psychological distress arising from the uncertainty around the permanence of disease (eg. inability to reconcile the need to take medication lifelong or fully embrace the diagnosis of T2D)[20].

Our study shows persons with T2D are often left with a feeling of “self under attack” when they are compelled to face sudden challenges in the form of behavioural modifications and dietary adaptation[9]. Evident from the details seen in this study, T2D people remain conflicted about the opposing needs to practice self-discipline and giving in to the inner craving to violate dietary restrictions[9,20].Thus for all of these transformations to occur smoothly, GPs should act as effective change agents in order to forge a collaborative relationship with individuals with T2D[19].

### Implications for future research and clinical implications

The narratives that transpired from these interviews dispel the “myth” that self-management practices are contingent on the actions of the people with T2D alone—the influence of their external environment, personal disposition and relevant behavioural mediators undoubtedly shape the course of their actions in equal measure[5,21]. Hence, healthcare professionals need to be wary that every T2D people present a unique set of challenges which can only be surmounted by customizing an individualized strategy for their disease management[22].

## Supporting information

S1 Table Relevant quotes describing self-management practices and the mediators of optimal self-care. (DOCX)

S2 Table Themes obtained via grounded theory approach (process coding). (DOCX)

S3 Table Consolidated criteria for reporting qualitative studies (COREQ): 32-item checklist. (DOCX)
