# Supplementary material for: The internal realities of individuals with type 2 diabetes – a functional framework of self-management practices via Grounded Theory approach
Source: PLoS One. 2019 Nov 26;14(11):e0225534. doi: 10.1371/journal.pone.0225534 (PMC6879143; doi:10.1371/journal.pone.0225534)
Supplement: S1 Table — (DOCX) [file pone.0225534.s001.docx]

S1 Table : Relevant quotes describing self-management practices and the mediators of optimal self-care

| **Themes** | **Definition** | **Quotes** |
| --- | --- | --- |
| ***Helpful***  Self Efficacy  Responsibility  Rationality  ***Unhelpful***  Restraint  Neglect  Experimentation | One’s ability to practice  proper disease management  Taking charge of disease and  taking steps towards proper  disease control  Forming logical, well-reasoned  point of views that contribute  to good disease control  Exercising poor volition in  terms of disease management  Disregard to proper disease  management practices  Trying various other  unsolicited means of  managing disease, leading to  poor disease control | I take my medication daily. So far, I have yet to miss any medication.  If I have two places that I frequently go to, I keep some medicine in both  those places, so I don’t have to worry about forgetting to bring my  medication along.  I have to comply with what the doctor prescribes. I believe that’s my role,  and I would like to assume that role accordingly.  I have disciplined myself to take my medication regularly.  I believe that the doctor knows best. What the doctor asks me to do, I will  follow through.  Those traditional medicine that they sell in the stores, to me, isn’t  effective.  Sometimes we think ‘if get diabetes, so be it’. For the moment, let’s just  consume whatever we want.  Sometimes, when I see the food lined up on the table, I get my cravings.  I’m only human, doctor.  Forgetting is something that happens to me. Personally, I think I’m quite  forgetful as a person.  Sometimes people tend to take it easy with their disease.  To tell you the truth, there is this medication that I was told to take two  tablets, but I only take one tablet.  I realize there was no change in my blood sugar levels. This was not due  to the doctor or the effectiveness of the medicine, I was toying the  dosage of the medications. |
