# Supplementary material for: The internal realities of individuals with type 2 diabetes – a functional framework of self-management practices via Grounded Theory approach
Source: PLoS One. 2019 Nov 26;14(11):e0225534. doi: 10.1371/journal.pone.0225534 (PMC6879143; doi:10.1371/journal.pone.0225534)
Supplement: S2 Table — (DOCX) [file pone.0225534.s002.docx]

**S2 Table. Grounded theory coding process of self-management practices**

| **Selective Coding** | **Axial Coding** | **Open Coding** | **Quotations** |
| --- | --- | --- | --- |
| Self-Management Practices  **Helpful**  *Positive actions taken to manage symptoms, treatment and lifestyle changes*  **Unhelpful**  *Negative actions taken to manage symptoms, treatment and lifestyle* | Self Efficacy | Complying to prescribed medication | “My children always see me taking my medicine regularly. So they know I automatically will take.” – **Male (IDM 017)**  “I really follow my medication as prescribed.” – **Female (IDM 019)**  “What the doctor has prescribed, I just follow.” – **Male (IDM 002)** |
|  |  | Bringing medication along when out of home | “If I happen to go outstation, I bring my medicine along with me.” – **Female (IDM 019)**  “Nowadays, the medicine is always in my bag.” – **Female (IDM 022)**  “I keep my medicine in a box, and I keep it with me at all times.” – **Male (IDM 010)** |
|  |  | Setting reminder on phone to improve compliance | “…Otherwise, what I do is I set a notification on my phone.” – **Male (IDM 018)**  “I use my phone as a reminder for my medicine.” – **Male (IDM 014)**  “My phone is an indicator that it’s time to take meds.” – **Male (IDM 010)** |
|  |  | Preventing missed dose of oral medications by storing in containers | “I keep all my medicine in a little box.” – **Male (IDM 010)**  “For myself, I keep my medicine in a small box…” – **Female (IDM 003)**  “I organize my medicine into little boxes…” – **Female (IDM 021)** |
|  |  | Making medication taking a routine activity | “The medicine is part of my life, doctor.” – **Male (IDM 013)**  “Medicine is a daily routine. We cannot forget.” – **Male (IDM 016)**  “If you are a routine kind of guy, then you will realize that something is missing if you don’t take your medicine.” – **Male (IDM 012)** |
|  |  | Reminding self to take medication | “When I forget my medicine, I will remind myself to take it later.” – **Male (IDM 010)**  “I keep reminding myself and try not to forget to take the medicine.” – **Female (IDM 003)**  “I keep telling myself I need to take the medicine properly.” – **Female (IDM 005)** |
|  |  | Adhering to proper timing when taking medications | “I have a timetable of when I should take the medicine.” – **Male (IDM 018)**  “I know that after I take my tablet, half an hour later, I have to have my meal. So I do that.” – **Female (IDM 020)**  “Medication, I try to be on time.” – **Male (IDM 017)** |
|  |  | Self-monitoring blood glucose levels | “I usually like to do that. Check my blood sugar before and after taking medicine.” – **Male (IDM 004)**  “I check my sugar maybe once a week or once every 2 weeks.” – **Male (IDM 012)**  “I check it (blood glucose levels) everyday. Every day, first thing in the mornings.” – **Male (IDM 008)** |
|  |  | Recalling proper insulin technique and utilization | “You have to change the needle regularly…” – **Male (IDM 009)**  “It’s (insulin) more effective if I take it in the stomach.” – **Female (IDM 006)**  “Need to shake before pressing…” – **Male (IDM 018)** |
|  |  | Practicing foot care | “I have to make sure my foot is clean, and I ask the doctor to check and make sure…” – **Male (IDM 002)**  “When I walk and all, whenever I do anything I am very careful about my feet.” – **Female (IDM 005)**  “I wear slippers indoors, because I’m afraid of small rocks and stones.” – **Female (IDM 024)** |
|  |  | Improving self care by using feedback from glucometer | “I check my own blood sugar, and draw motivation from that.” – **Male (IDM 018)**  “When I check my sugar level, and it goes up to about 9 or 10, then I will do something to bring it down.” – **Male (IDM 010)**  “I will go and check, and when it is high, I will backtrack and question what I have eaten or done to cause it to be so high.” – **Female (IDM 011)** |
|  |  | Following up for T2D check ups | “And so, till this day I continue going for all my check-ups.” – **Male (IDM 018)**  “I have check-ups that I follow regularly.” – **Female (IDM 011)**  “I still follow my 4 months once control visits here (at the GP’s clinic). – **Female (IDM 005)** |
|  |  | Needing to consult doctor if in doubt | “I’m afraid to change the dose on my own. So I’ll ask the doctor. I don’t have that knowledge. The doctor does.” – **Male (IDM 017)**  “I found out that all these other remedies are dangerous. In order to cure the disease, you need the doctor…” - **Male (IDM 013)**  “Whenever something small happens, I always say, go to the doctor and get a check-up.” – **Male (IDM 001)** |
|  |  | Preventing complications by taking medications | “The two complications I’m most worried about is kidney failure and stroke. So by taking this medication, I hope that I can avoid these two.” – **Male (IDM 017)**  “As a diabetic patient we have to take our medicine. If we don’t do it now, later on we’ll face all sorts of problems.” – **Male (IDM 013)**  “I believe that medicine helps me control my sugar. If I control my sugar, everything will be alright. I won’t get any complications.” – **Male (IDM 002)** |
|  |  | Needing to control diet | “We must control our diet.” – **Male (IDM 001)**  “Food intake you must control.” – **Male (IDM 017)**  “Nowadays I really control my diet. I avoid all those things that I’m not supposed to eat.” – **Male (IDM 023)** |
|  |  | Following dietary advice | “I’m implementing changes in my diet.” – **Female (IDM 003)**  “I’ve drastically reduced the sugar I take in.” – **Female (IDM 006)**  “I know how to control my food and keep myself under control.” – **Female (IDM 005)** |
|  |  | Reducing the intake of rice | “When I eat out I ask them to put less rice for me.” – **Female (IDM 003)**  “…or I take a very small quantity of rice.” – **Female (IDM 005)**  “I reduce my rice intake. For example, if I take one meal of rice, my next meal I don’t take rice. I take something else.” – **Male (IDM 016)** |
|  |  | Choosing more vegetables | “Nowadays I have developed a preference towards raw vegetables, like salads and whatnot.” – **Female (IDM 019)**  “I control my food by taking less rice and more vegetables.” – **Female (IDM 020)**  “I eat vegetables, it is good for me.” – **Female (IDM 011)** |
|  |  | Using dietary measures to bring down elevated blood glucose | “I control my food to control my sugar level.” – **Male (IDM 012)**  “I control my food. I don’t simply eat anything. And when I check my blood, it’s 6, or 5.5.” – **Male (IDM 010)**  “I told myself to cut out all the sugary foods, so that I can bring my body back to health.” – **Male (IDM 013)** |
|  |  | Wishing to bring down blood glucose to optimal level | “I want to bring my blood sugar down. I want it to be 5 or 6.” – **Male (IDM 014)**  “I feel that I can really control it, and I want to bring my sugar down from it’s current level.” – **Male (IDM 017)**  “My blood sugar is 7.7. I wish to reduce it further.” – **Male (IDM 008)** |
|  |  | Exercising to control blood glucose | “I try to reduce the sickness by doing exercise.” – **Female (IDM 003)**  “As of late I’ve started to do a little jogging, and it’s brought down my sugar to 7.7.” – **Male (IDM 008)**  “At home I exercise and feel quite good.” – **Male (IDM 018)** |
|  | Rational | Trusting information from internet | “In my experience, some of the things that influence my diabetes are articles that I read on the internet.” – **Male (IDM 015)**  “See that’s the thing, doctor. Because I don’t have the knowledge, I’ve got to explore. Read articles on the internet. And so I read these things on the internet, articles written by medical websites or other doctors.” – **Male (IDM 023)**  “So I just type the name of the medication in the internet and I can see everything about it.” – **Male (IDM 001)** |
|  |  | Choosing what information is useful to us | “When people tell me things, it’s not that I accept everything. I decide what to accept.” – **Female (IDM 011)**  “When they (people) tell you something, you need to think for yourself, whether it can be accepted or not.” – **Male (IDM 016)**  “I listen to the information, but I see first. I balance everything, and decide which I want to follow.” – **Female (IDM 020)** |
|  |  | Believing evidence based information | “Newspapers always come with the latest information, doctor. Information about researches done overseas and everything.” – **Male (IDM 002)**  “I educate myself and do my own research.” – **Male (IDM 014)** |
|  |  | Being skeptical about CAM | “If it isn’t western medicine, that means it (CAM) comes from leafs and herbs. And there’s no proof that it works.” – **Male (IDM 010)**  “Traditional medicine, as in the products sold out there? To me that’s rubbish.” – **Female (IDM 021)**  “Those village remedies, I strictly don’t take.” – **Female (IDM 011)** |
|  |  | Dismissing the need for CAM when conventional management effective | “When the medicine I’ve been given works, I don’t see why I need to go and take anything else.” – **Female (IDM 021)**  “When I think about it, it’s much better for me to follow the medication provided by the hospitals.” – **Male (IDM 015)**  “Traditional medicine is useless. You’re much better off with medicine from the hospital.” – **Male (IDM 007)** |
|  |  | Being suspicious about CAM leading to side effects | “I also don’t know whether taking all these (CAM) will lead to side effects or not.” – **Male (IDM 010)**  “Traditional medicine, we only have word of mouth to go by. But if anything happens, that’s it.” – **Female (IDM 006)**  “No, traditional medicine should not be consumed. You never know, it could cause you heart problems and you’ll suffer.” – **Male (IDM 007)** |
|  |  | Being skeptical about efficacy of CAM in self | “I don’t know whether the traditional medicine I’m taking works on me or not.” – **Male (IDM 004)**  “I used to take for a few months, but I didn’t feel any difference at all.” – **Male (IDM 023)**  “I tried it and I felt my body didn’t respond well to me. So I stopped it.” – **Female (IDM 024)** |
|  |  | Trusting doctor's advice or instruction | “The guy who decides should be the doctor. He’s the person that knows best.” – **Male (IDM 008)**  “What the doctor says, I’ll do. I will follow the doctor’s procedure.” – **Female (IDM 019)**  “I will take my medication according to the advice of the doctor.” – **Male (IDM 017)** |
|  |  | Knowing it is up to us whether to accept comments of others | “People tend to say a lot of different things, but it’s up to us whether we listen to it or not.” – **Male (IDM 004)**  “People say all sorts of things; try to get me to do all sorts of things, none of that influences me.” – **Female (IDM 005)**  “My friends say a lot of things, telling me I’m weak or I have a weakness. None of that influences me.” – **Male (IDM 016)** |
|  |  | Trusting doctor's advice is completely up to patient | “If the patient wants to take the medicine, he’ll take it. If he doesn’t want to take it, he won’t. The patient will choose.” – **Female (IDM 020)**  “For me it’s not an issue. The advice, if I can follow, I follow. If I can’t follow, I don’t follow.” – **Male (IDM 009)**  “Whether or not the patient is willing to accept the advice is up to them.” – **Male (IDM 018)** |
|  | Dietary restraint | Restricting sugar intake | “I’ll tell them to put less sugar in my tea.” – **Male (IDM 002)**  “Nowadays when I order my tea I immediately ask for less sugar, or no sugar.” – **Male (IDM 012)**  “I am not taking sugar anymore. For my drinks I’m using sweeteners.” – **Female (IDM 003)** |
|  |  | Being able to still enjoy food by reducing amount previously consumed | “So these days I take my meds, and I eat what I want. But not a lot. In small amounts, just to taste.” – **Female (IDM 024)**  “Everyone asks whether I can take some sweet things and all. I can. For example, if I go to a birthday party, I’ll take the cake, but only a very small piece.” – **Female (IDM 020)**  “I had to change my entire diet. Certain things I can’t take, some I take in smaller amounts.” – **Female (IDM 011)** |
|  |  | Practicing restraint when consuming food | “You can choose what you want to eat. You don’t have to put all the junk into your mouth.” – **Female (IDM 003)**  “When there are gathering, I politely refuse, take my plain water and a small amount of food, and eat.” – **Male (IDM 023)**  “I really control my diet. Vegetables and fish. I controlled with that type of food.” – **Female (IDM 019)** |
|  |  | Avoiding sugary beverages | “I don’t have the urge to take sugary drinks anymore.” – **Male (IDM 012)**  “I tell the people who are unaware that I’m diabetic, not to give me sweet drinks.” – **Female (IDM 020)**  “I don’t drink carbonated drinks.” – **Male (IDM 017)** |
|  | Responsibility | Being proactive about disease | “I actively learn, maybe by asking a lot of questions.” – **Male (IDM 004)**  “I do research for myself about insulin and all that.” – **Male (IDM 014)**  “Each time the doctor changes or increases my medication; I’ll make it a point to ask him about the drug I’m taking.” – **Female (IDM 011)** |
|  |  | Knowing discipline is important in T2D management | “You have to be disciplined to take the medicine properly. Then you will see the results.” – **Male (IDM 012)**  “I am a person with certain principles. If this is something I have to do, I do it. No matter what.” – **Female (IDM 011)**  “We have to train ourselves, and really take a lot of effort to learn to comply to the medicine.” – **Male (IDM 013)** |
|  |  | Knowing its patient's responsibility to comply with medication | “It’s my duty to take the medicine.” – **Male (IDM 016)**  “If we are responsible to ourselves, we’ll take the medicine as per regulation.” – **Male (IDM 015)**  “We need to take that medicine, we need to be responsible…” – **Female (IDM 021)** |
|  |  | Taking responsibility to look after their health | “You have to be serious (about health) now that you have diabetes…” – **Male (IDM 008)**  “I have to take care of myself.” – **Male (IDM 002)**  “It is definitely the individual who’s responsible for their disease.” – **Male (IDM 012)** |
|  |  | Maintaining health by taking medication | “I take the disease very positively. The most important thing is to take the medicine to control my blood sugar.” – **Male (IDM 008)**  “If I want to survive, I have to continue taking medicine.” – **Male (IDM 002)**  “I take my medication to keep my sugar levels low.” – **Male (IDM 010)** |
|  |  | Maintaining health so that can live longer | “One of the reasons I do this (control) is because I think of the long run.” – **Male (IDM 018)**  “I follow the doctor’s advice, and if I’m healthy, then I can stay a little longer here (on earth).” – **Male (IDM 002)**  “If I die, I die. But I try to prolong my life. To me that’s good enough already.” – **Male (IDM 017)** |
|  |  | Staying healthy to improve quality of life | “If I want my legs, fingers and eyes to be fine, I have to take my medicine. That’s all.” – **Female (IDM 005)**  “Everyone wants a good, healthy life. And I have this diabetes. If I don’t control it, I could go blind…” – **Male (IDM 014)**  “We try to control because we want to be better. Otherwise we will be very sick.” – **Female (IDM 003)** |
|  |  | Being healthy so can support the family | “I need to work on my health because I need to take care of my family.” – **Male (IDM 002)**  “I don’t want my child to be motherless.” – **Female (IDM 006)**  “My generation tends to think this way. We think that we have to be strong for our family. So if we have any sickness, we would take the medicine, because we have a responsibility (to family).” – **Female (IDM 021)** |
|  |  | Managing disease independently | “I manage my own disease (no involvement of family members).” – **Male (IDM 012)**  “No my family doesn’t need to prompt me to take medication.” – **Male (IDM 010)**  “My family, my children don’t advise me because they don’t know enough about diabetes.” – **Male (IDM 023)** |
|  |  | Maintaining health to avoid being a burden to others | “I must take care of my disease so that I won’t be a burden to my family and my people.” – **Male (IDM 002)**  “I don’t want to trouble my children to have to send me to and fro from the hospital.” – **Male (IDM 015)**  “If I don’t take the medicine, I’m troubling my family members.” – **Male (IDM 013)** |
|  |  | Persevering with medication despite side effects | “Even today the side effects are there. But I’m not bothered.” – **Female (IDM 011)**  “Even though the metformin gives me trouble, I still continue to take it, because the doctor asked me to.” – **Female (IDM 006)**  “I feel all these (side effects), but I still take my medicine. I never stop. I don’t want to stop.” – **Female (IDM 005)** |
|  |  | Complying to medication despite no physical symptoms | “Yes, I believe we should take our medicine and come for check-ups, even if we feel physically well.” – **Male (IDM 002)**  “Even though I feel really healthy, I’ll still take my insulin.” – **Male (IDM 006)**  “Even if I feel healthy, I’ll continue with the medication.” – **Male (IDM 017)** |
|  |  | Missing medications infrequently | “…but about 90% of the time, I don’t miss my medicine.” – **Female (IDM 011)**  “I have forgotten before, but it’s a very rare occurrence.” – **Female (IDM 006)**  “Once in a while I’ll forget.” – **Male (IDM 009)** |
|  | Neglect  Poor Restraint | Missing medication because of procrastination | “Why I miss my medicine? I’m not sure if this is the right word for it, procrastination? Yeah.” – **Male (IDM 010)**  “I put off taking medicine when I should, then later on, when I should take, I’ll be on an empty stomach. Then I don’t know whether to take or not.” – **Female (IDM 011)**  “Maybe sometimes people feel that they don’t need to take the medicine today, I’ll take it tomorrow…” – **Female (IDM 020)** |
|  |  | Confessing having skipped medications | “There have been times where I don’t take my medicine.” – **Male (IDM 004)**  “I’m also not that good at complying with my medicine.” – **Male (IDM 015)**  “Previously I never used to take my medicine.” – **Male (IDM 007)** |
|  |  | Attributing forgetfulness to take medications to old age | “I forget sometimes. You know, especially us seniors, we’re prone to forget.” – **Male (IDM 018)**  “Forgetfulness is an issue (in taking medication). Especially amongst people who are older.” – **Female (IDM 011)**  “People who get older are bound to forget their medications.” – **Male (IDM 016)** |
|  |  | Reminding self to take medication as appointment approaches | “The closer you come to the appointment date, you will start to worry and start taking your medicine.” – **Male (IDM 012)**  “When it’s nearer the appointment date, only then will I start taking my medicine properly, because I don’t want to get scolded by the doctor.” – **Male (IDM 015)**  “If I’m being honest, there was a time where I would only control when it was close to the appointment date.” – **Female (IDM 019)** |
|  |  | Having no awareness about the dangers of high sugar diet | Those days, I used to eat a lot of sweets in India. For 14 years. There was no awareness at all about diabetes back then.” – **Male (IDM 001)**  “It was a habit formed in my younger days. Staying overseas, I drank Coca-Cola 100% of the time.” – **Male (IDM 023)**  “Coca-Cola used to be a daily affair for me. Back then I didn’t know about the effects of such drinks.” – **Male (IDM 018)** |
|  |  | Confessing not doing much exercise | “For me, I don’t exercise.” – **Male (IDM 018)**  “I don’t exercise very much.” – **Male (IDM 010)**  “The thing about me is that I hardly do exercise.” – **Female (IDM 024)** |
|  |  | Missing medication due to forgetfulness | “Sometimes I forget. I leave the pill box at home.” – **Male (IDM 008)**  “I rarely miss, unless, for example, certain days I forget.” – **Female (IDM 019)**  “I almost always take it. Unless I forget.” – **Female (IDM 022)** |
|  |  | Having lackadaisical attitude towards T2D management | “There are many reasons why diabetes is a norm. For example the craving for food and the ‘don’t care’ attitude.” – **Female (IDM 011)**  “I take the medicine from the doctor, but when I go home, I go back to my old lifestyle…” – **Male (IDM 015)**  “You can control your disease by taking medication, dieting and exercising regularly. However, I don’t do it.” – **Male (IDM 009)** |
|  |  | Missing or skipping medication (deliberate) | “I didn’t take that medicine at all, because I know my health was okay.” – **Male (IDM 013)**  “Some medicine I’ve been asked to take daily, I’ll take once every two days.” – **Male (IDM 010)**  “Just like that, one day I could not take it anymore. I didn’t want to take any of my medicine. I went like that for two days.” – **Female (IDM 011)** |
|  |  | Violating dietary restriction | “I don’t follow the doctor’s advice especially when it comes to food.” – **Male (IDM 002)**  “My diabetes isn’t very well controlled, mostly due to my diet.” – **Male (IDM 015)**  “In the middle of the night, when I’m driving my lorry, if I feel sleepy I’ll eat whatever’s convenient, mostly stall food.” – **Male (IDM 007)** |
|  |  | Being ignorant about the disease | “When I was diagnosed with diabetes, I knew I had a disease, but I knew nothing about the disease.” – **Female (IDM 024)**  “The doctors told me I had diabetes, but I didn’t understand. To me, I was healthy.” – **Male (IDM 013)**  “I didn’t know anything about this disease, and I didn’t understand anything. I didn’t bother going out there to find out ether.” – **Female (IDM 021)** |
|  |  | Failing to take medication according to proper timing | “I take my medication. It’s just that sometimes, due to my lifestyle, the timing tends to be erratic.” – **Male (IDM 004)**  “For example, I am supposed to take a tablet in the morning, but sometimes I don’t have my breakfast, and so I forget about the medication. Then later when I take it, it isn’t according to the proper time.” – **Female (IDM 021)**  “Sometimes I sleep through the time I’m supposed to take medicine. Now I’m not working, so my sleeping hours are irregular.” – **Male (IDM 013)** |
|  |  | Blaming self for lapses in T2D management | “Sometimes, it’s my mistake. I don’t follow the doctor’s advice. Especially in regards to food.” – **Male (IDM 002)**  “I feel that, if ever I were to get any complications, like my foot getting amputated, for example, it would be my fault. It would be because I didn’t control properly.” – **Male (IDM 023)**  “Nobody influences my medicine intake. If I fail to take my medicine, then that’s my own fault.” – **Female (IDM 005)** |
|  |  | Confessing having missed medication | “During my younger days, I never used to take my medicine. I always missed it.” – **Female (IDM 006)**  “To be frank, I miss my medicine as well sometimes. Especially at night, when I have to go somewhere.” – **Female (IDM 022)**  “Yeah as I’ve mentioned, I sometimes forget to take my medicine.” – **Female (IDM 019)** |
|  |  | Being unaware of having T2D | That time, if I had not gotten dengue and gotten my tests, I would not have known I had diabetes.” – **Male (IDM 002)**  “I only discovered I had diabetes when I went for my check-up. I wanted to attend a course to learn sales, so the requirement was to get a medical check-up done.” – **Male (IDM 007)**  “Me discovering I had diabetes was coincidental. I came in for my back pain. Only then did I realize I had hypertension and diabetes.” – **Female (IDM 005)** |
|  |  | Lacking discipline to follow T2D management advice | “Believing (diabetes and methods to control) is one thing. Actually performing it is another thing altogether.” – **Male (IDM 010)**  “Sometimes because of greed, I tend to eat extra.” – **Female (IDM 011)**  “It’s just that, as a diabetic patient, I’m not very disciplined.” – **Female (IDM 024)** |
|  |  | Complying to T2D management once complications set in | “I only started taking my diabetes seriously after I got married. My eyesight had become very bad. I could not see.” – **Female (IDM 006)**  “My own wife only started taking her medicine after her foot got gangrene.” – **Male (IDM 023)**  “It was only that time when I was admitted for my carbuncle did I start controlling well.” – **Female (IDM 024)** |
|  |  | Thinking about not complying due to no physical symptoms | “Yes, during the initial stages of my disease, I too thought that I didn’t have to take medicine once I felt healthy.” – **Male (IDM 004)**  “Sometimes I try not to think about it. If I feel healthy, then I just enjoy myself, and I think that helps.” – **Male (IDM 007)**  “I tend to only control when I feel sick. When I feel healthy, I go back to my daily routine.” – **Female (IDM 024)** |
|  |  | Realizing T2D too late when extreme symptoms arise | “I didn’t know I had the disease until I started feeling really sick and weak.” – **Male (IDM 018)**  “That time, I felt really giddy and thirsty. Then my boss’s brother asked me to go check my sugar. And then I was diagnosed with diabetes.” – **Male (IDM 002)**  “The thing about diabetes is that you can’t really feel it until much later on when you start to feel really ill.” – **Female (IDM 022)** |
|  |  | Violating proper insulin technique | “If you don’t change the insulin needle regularly, when you poke, it feels very painful.” – **Male (IDM 009)**  “They advised me to regularly change my insulin needles, but, sorry to say doc, I don’t really do that.” – **Female (IDM 024)**  “Back then, I didn’t take my insulin properly. I never shake the bottle, and I never used to press first before injecting.” – **Male (IDM 018)** |
|  |  | Failing to disclose clinical information about self to doctor | “I know it’s my fault, but sometimes I don’t really tell the doctor things about my disease…” – **Male (IDM 004)**  “Sometimes we don’t tell the doctor everything because we are afraid the doctor may scold us.” – **Female (IDM 003)**  “I faced all these issues, but I’ve never really told the doctor. I know the doctor has his own job, and he will advise me to take the medicine. That’s fixed. So I’ll follow what I can.” – **Male (IDM 007)** |
|  |  | Detecting predominantly high blood glucose levels with glucometer | “Most of the time my glucose is really high when I test it.” – **Male (IDM 004)**  “Every time I check my (blood glucose) readings, the results are bad.” – **Female (IDM 005)**  “When I come and check it, the readings are almost always high.” – **Female (IDM 019)** |
|  |  | Attributing T2D to uncontrolled food consumption | “My diet isn’t very good, doctor. I tend to eat a lot of ice creams. And for dinner I usually drink sugary drinks.” – **Female (IDM 019)**  “Nowadays we have all the junk food and fast food that cause this disease.” – **Male (IDM 001)**  “As Malaysians, we are sugar lovers. We overindulge in sugar. So in the long run, we will reap the effects of our bad habits.” – **Male (IDM 012)** |
|  |  | Frustrating having to follow diet restrictions | “Food restrictions are one of the main reasons why diabetes control fails. When a normal person can eat that food, why can’t I? I didn’t ask for this disease.” – **Male (IDM 012)**  “If you are a person who likes to eat, then controlling this disease is really hard. When I go to a function, I feel so restricted. There is so much good food, but I can’t eat.” – **Male (IDM 010)**  “Of course the doctor is right in saying all those things that we are not supposed to eat. But it sometimes just stresses me out.” – **Male (IDM 023)** |
|  |  | Expressing difficulty in controlling diet | “I feel that controlling the diet is really difficult.” – **Male (IDM 002)**  “When you sit down with the dietician, all the advice the dietician gives you is good and for your own good. But sometimes it’s just not practical.” – **Male (IDM 012)**  “Controlling my diet is difficult, doctor. Especially since, since young, I’ve been raised to eat heavy food. Now you’re telling me to change and eat a few slices of bread and oats? I can’t even sleep because of that.” – **Male (IDM 015)** |
|  |  | Violating dietary restrictions due to social pressure | “I try to inform them (friends/guests) in advance about my diabetes. However if they’ve given me the food or sugary drink, I cannot refuse ether.” – **Female (IDM 020)**  “Then I’ll make a stop on the way to deliver goods, and have a drink with my friends, and drink and eat junk food.” – **Male (IDM 007)**  “The food is the difficult part. When 2 or 3 of us gather at the hawker stalls, it’s really difficult to maintain dietary control.” – **Male (IDM 015)** |
|  |  | Consuming forbidden or excess food despite knowing | “I’ve spoken to the dietician and I know about the portions I should be eating. But sometimes, I just feel hungry, doctor. And when that happens, I’ll eat till I’m full.” – **Female (IDM 019)**  “Sometimes, because of greed, we eat more than we should.” – **Female (IDM 011)**  “Sometimes, when we’re gathered with family, and all the food is laid out before us, we get tempted and we give in.” – **Male (IDM 023)** |
|  |  | Condoning violations in dietary restrictions (infrequent) | “I can control well for most things. I take my medicine and I cut down all sugary stuff. But the things I cannot give up are rice and coffee.” – **Female (IDM 006)**  “Once in a while, doctor, when we eat out, the temptation is there. So I indulge.” – **Male (IDM 017)**  “At times I binge eat. I cannot really control myself.” – **Male (IDM 008)** |
|  | Experimentation | Changing and continuing medication dose (not as prescribed) | “I’ve done some experiments with my medicine. And I’ve concluded that I’ll keep taking my medicine, but at a lesser dose.” – **Male (IDM 004)**  “To tell you the truth, this one medicine, doctor prescribed 2, I only take 1 and a half.” – **Male (IDM 010)**  “Before this, I have tried to lower my dose. I’ve never been brave enough to try increasing it.” – **Female (IDM 021)** |
|  |  | Seeing increase in blood glucose levels after altering/skipping dose | “I realized that I should not experiment with my medication, when I saw that my blood sugar was not improving.” – **Male (IDM 012)**  “So I play around with the dose and sometimes skip medicine for a day. Then when I check, I see an increase in my sugar levels.” – **Female (IDM 011)**  “When I take the full 500mg I feel dizzy. So I reduce the amount. Then when I meet with my doctor, my readings all go up.” – **Male (IDM 007)** |
|  |  | Taking CAM | “I take some traditional medicines, like herbs.” – **Male (IDM 004)**  “I used to go to India once a year. When I’m there I take the Indian traditional medicine.” – **Male (IDM 002)**  “What I do is I chew on this ‘neem’ leaf.” – **Male (IDM 012)** |
|  |  | Setting time interval for consuming CAM after medications | “There is no harm in taking traditional medicines, but you must leave a gap between the hospital medicine and the herbal medicine.” – **Male (IDM 001)**  “Yes if I take my herbal medicine, I’ll wait one hour before taking my normal medicine.” – **Male (IDM 016)**  “If you take traditional medicine you must leave a gap of at least half an hour before taking your hospital medicine.” – **Male (IDM 009)** |
|  |  | Adding CAM to current treatment (not replacing) | “So I take this traditional medicine, more to supplement the treatment.” – **Male (IDM 004)**  “When taking traditional medicine, you should not stop the hospital medicine. You must take both.” – **Male (IDM 002)**  “No, I’m not replacing my western medicine. I take cinnamon and I take my normal medicine.” – **Female (IDM 020)** |
|  |  | Replacing medication with CAM (entirely - skip) | “I once tried, for a period of time, to abandon my modern medicine and take herbal medicine.” – **Male (IDM 004)**  “I replaced metformin with herbal medicine, because metformin gave me some sort of giddiness.” – **Male (IDM 013)**  “Sometimes I’ll try the remedies my friends give me. I’ll take the traditional medicine exclusively for a few days.” – **Male (IDM 015)** |
