# Supplementary material for: The internal realities of individuals with type 2 diabetes – a functional framework of self-management practices via Grounded Theory approach
Source: PLoS One. 2019 Nov 26;14(11):e0225534. doi: 10.1371/journal.pone.0225534 (PMC6879143; doi:10.1371/journal.pone.0225534)
Supplement: S3 Table — (DOCX) [file pone.0225534.s003.docx]

**S3 Table. Consolidated criteria for reporting qualitative studies (COREQ): 32-item checklist**

| **No. Item** | **Guide questions/description** | **Reported on Page #** |
| --- | --- | --- |
| **Domain 1: Research team and reﬂexivity** |  |  |
| *Personal Characteristics* |  |  |
| 1. Inter viewer/facilitator | Which author/s conducted the inter view or focus group? | Page 3 |
| 2. Credentials | What were the researcher’s credentials?E.g. PhD, MD | Title Page |
| 3. Occupation | What was their occupation at the time of the study? | Title Page |
| 4. Gender | Was the researcher male or female? | Title Page |
| 5. Experience and training | What experience or training did the researcher have? | Title Page |
| *Relationship with participants* |  |  |
| 6. Relationship established | Was a relationship established prior to study commencement? | Page 3 (refer to published study protocol)  . |
| 7. Participant knowledge of theinterviewer | What did the participants know about the researcher? e.g. personal goals, reasons for doing theresearch | Page 3 (refer to published protocol) |
| 8. Interviewer characteristics | What characteristics were reported about the inter viewer/facilitator? e.g.Bias, assumptions,reasons and interests in the research topic | Page 3 (refer to published protocol) |

| **Domain 2: study design** |  |  |
| --- | --- | --- |
| *Theoretical framework* |  |  |
| 9. Methodological orientation andTheory | What methodological orientation was stated to underpin the study?e.g. grounded theory,discourse analysis, ethnography, phenomenology, content analysis | Page 3 –4 |
| *Participant selection* |  |  |
| 10. Sampling | How were participants selected?e.g. purposive, convenience, consecutive, snowball | Page 3 |
| 11. Method of approach | How were participants approached? e.g. face-to-face, telephone, mail, email | Page 3 |
| 12. Sample size | How many participants were in the study? | Page 3 |
| 13. Non-participation | How many people refused to participate or dropped out? Reasons? | Page 3 |
| *Setting* |  |  |
| 14. Setting of data collection | Where was the data collected? e.g. home, clinic, workplace | Page 3  . |
| 15. Presence of non-participants | Was anyone else present besides the participants and researchers? | Page 3 |
| 16. Description of sample | What are the important characteristics of the sample?e.g. demographic data, date | Tables 1,2 (also refer to published study protocol) |
| *Data collection* |  |  |
| 17. Interview guide | Were questions, prompts, guides provided by the authors? Was it pilot tested? | Page 43(refer to published protocol and submitted paper) |
| 18. Repeat interviews | Were repeat inter views carried out? If yes, how many? | No, inferred on page 3 - 4 |
| 19. Audio/visual recording | Did the research use audio or visual recording to collect the data? | Page 3 |
| 20. Field notes | Were ﬁeld notes made during and/or after the inter view or focus group? | Page 3 (refer to published protocol) |
| 21. Duration | What was the duration of the inter views or focus group? | Page 3 (refer to published protocol) |
| 22. Data saturation | Was data saturation discussed? | Yes, inferred on page 3 |
| 23. Transcripts returned | Were transcripts returned to participants for comment and/or correction? | No, but audio recording and transcripts were proofread by an independent researcher to confirm its validity(refer to published protocol) |
| **Domain 3: analysis and ﬁndings** |  |  |
| *Data analysis* |  |  |
| 24. Number of data coders | How many data coders coded the data? | Page 3 |
| 25. Description of the coding tree | Did authors provide a description of the coding tree? | Table 3, Figure 1 |
| 26. Derivation of themes | Were themes identiﬁed in advance or derived from the data? | Page 2, Page 3 (refer to published protocol) |
| 27. Software | What software, if applicable, was used to manage the data? | Page 3 – 4 |
| 28. Participant checking | Did participants provide feedback on the ﬁndings? | Page 3 – 4 (yes, during focus group discussions) |
| *Reporting* |  |  |
| 29. Quotations presented | Were participant quotations presented to illustrate the themes/ﬁndings? Was each quotation identiﬁed? e.g. participant number | Page 4 to 9 |
| 30. Data and ﬁndings consistent | Was there consistency between the data presented and the ﬁndings? | Yes, there was.  Page 4 to 10 |
| 31. Clarity of major themes | Were major themes clearly presented in the ﬁndings? | Yes. Page 4 – 10 and Table 3 + Figure 1 |
| 32. Clarity of minor themes | Is there a description of diverse cases or discussion of minor themes? | Page 9 to 10 |

*Research protocol

Swarna Y, Haque S. The development of an integrated behavioural model of patient compliance with diabetes medication : a mixed-method study protocol. Fam Pract. 2018;(1):1-6. doi:10.1093/fampra/cmy119
